# Assessment of the masseter stiffness in patients during conservative therapy for masticatory muscle disorders with shear wave elastography

**DOI:** 10.1186/s12891-022-05392-9

**Published:** 2022-05-11

**Authors:** Anna Olchowy, Piotr Seweryn, Cyprian Olchowy, Mieszko Wieckiewicz

**Affiliations:** 1 Department of Experimental Dentistry, Wroclaw Medical University, 50-425 Wroclaw, Poland; 2 Department of Oral Surgery, Wroclaw Medical University, 50-425 Wroclaw, Poland

**Keywords:** Masseter, Shear wave elastography, Temporomandibular disorders, Occlusal splint, Massage, Masticatory muscle disorders

## Abstract

**Background:**

The complex structure of the stomatognathic system plays a vital role in chewing, digestion, speaking, breathing, facial expression and swallowing. Its complexity is the primary reason for creating multidisciplinary teams to manage temporomandibular disorders (TMD). We aimed to assess the masseter stiffness in patients undergoing conservative therapy for masticatory muscle disorders and evaluate the efficacy of manual therapy and stabilization occlusal splint in the treatment of masticatory muscle disorders.

**Methods:**

This uncontrolled prospective cohort study included 35 patients with masticatory muscle disorders. The study lasted for eight weeks. The patients were treated with manual therapy and stabilization occlusal splint and evaluated using shear wave elastography of the masseter muscles and patient-reported outcome measures (PROMs) to assess pain, anxiety, quality of sleep, satisfaction with life and perceived stress.

**Results:**

After the treatment, the stiffness of both masseter muscles decreased significantly (by 4.21 kPa). The patients reported a significant reduction in pain. At baseline, the median scores ranged from 5 to 8; after treatment, they ranged from 0 to 1 (*p* < 0.0001). The patients also reported significant improvement in terms of all patient-reported outcome measures. The reduction in stiffness corresponded to the improvement in pain and PROMs, as shown by correlations which were insignificant for all measures.

**Conclusions:**

Conservative therapy of masticatory muscle disorders involving manual therapy and stabilization occlusal splint is effective. It reduces the masseter stiffness as objectively shown in shear wave elastography and improves subjective PROMs scores, including numerical pain assessment and selected questionnaires. Shear wave elastography has the potential for broad application in clinical practice to monitor masticatory muscle disorders treatment effects due to its objectivity and non-invasive character. Further research is recommended on larger patient populations and longer follow-up.

**Trial registration:**

The study was registered at clinicaltrials.gov (NCT03844854). First posted date: 19/02/2019.

**Supplementary Information:**

The online version contains supplementary material available at 10.1186/s12891-022-05392-9.

## Background

The stomatognathic system plays a vital role in chewing, digestion, speaking, breathing, facial expression and swallowing. In addition, its complex structure requires harmonious cooperation of skeletal structures (maxilla and mandible), soft tissues, temporomandibular joints and masticatory muscles [1]. This complexity is one of the reasons for creating multidisciplinary teams to manage frequently reported temporomandibular disorders (TMD).

The Diagnostic Criteria for Temporomandibular Disorders (DC/TMD) represent a comprehensive approach to TMD diagnosis [2]. TMD patients may present with disorders in the masticatory muscles, which control the mandible movements and temporomandibular joints. Other symptoms include sounds and/or pain in temporomandibular joints (clicking and/or popping and/or crepitus), locking and limited movement of the mandible [3]. In addition, the patients may also experience symptoms that appear unrelated to the stomatognathic system, such as headache, neck and/or shoulder pain, or otolaryngological problems (e.g. tinnitus) [1, 4].

The prevalence of TMD in the Polish urban population is estimated at 55.9% [5]. The preliminary assessment can be performed by a general dentist and includes an assessment of the face and occlusal arch symmetry, palpation of masticatory muscles and temporomandibular joints, investigation of the entire dentition for number and position of teeth along with their surfaces [6]. Patients are first referred to conservative treatment, and next to surgery in the case of failure. Conservative treatment strategies aim to reduce pain and disability caused by TMD. A wide variety of techniques are used, including manual soft tissue work, splint therapy and electrophysical modalities [7]. However, TMD treatment is long and may require the participation of a more qualified dentist, physiotherapist, maxillofacial surgeon and other specialists [8]. In addition, even minor abnormalities in oral structure and function may reduce the patient quality of life [6]. Low TMD awareness [9, 10], long-term treatment and the lack of objective measures make the TMD treatment challenging.

Technological advances in diagnostic methods allowed the introduction of shear wave elastography to diagnose and monitor the treatment of masticatory muscles disorders. This method enables measuring the stiffness of soft tissues. Shear waves are generated by pressure and then detected by longitudinal ultrasonography waves that propagate in tissues significantly faster than shear waves. This method is non-invasive and shows high intra- and inter-operator agreement, high repeatability and the possibility to provide quantitative results [11, 12]. The body of evidence on the use of shear wave elastography in dentistry is growing [13]. It was validated against phantoms of known hardness [14, 15] and compared with other methods [16]. The results of shear wave elastography were proved reliable and reproducible [17, 18]. Furthermore, the literature reports suggest that this tool is reliable in measuring the changes in stiffness of the masseter muscle in response to physical therapy (namely massage) [19, 20] and exercise [21], both of which may be used in TMD management. However, there is little evidence on the use of shear wave elastography to evaluate treatment effects in patients undergoing conservative treatment for masticatory muscle disorders. With this gap in the current knowledge in mind, we performed this study among patients suffering from masticatory muscle disorders evaluating their treatment with patient-reported outcome measures (PROMs) along with an objective assessment of the masseter muscles using shear wave elastography.

### Objectives

The primary objective of this uncontrolled prospective cohort study was to assess the masseter stiffness in patients after a conservative therapy for masticatory muscle disorders using shear wave elastography. The secondary aim of this study was to evaluate the efficacy of manual therapy and stabilization occlusal splint in the treatment of masticatory muscle disorders. For this purpose, we investigated whether the objective measure of masseter muscle stiffness corresponded to subjective improvement in PROMs, including numerical pain assessment and selected questionnaires.

## Methods

### Participants

Overall, 38 people were examined of which 35 people meet inclusion criteria and were included in the study. Included patients were referred for the baseline visit directly. They gave informed written consent before the study and agreed to participate in all the study procedures. The study was approved by the Bioethics Committee at the Wroclaw Medical University (KB – 592/2018). The study was first posted on 19/02/2019 at clinicaltrials.gov (NCT03844854).

To calculate the sample size, we have taken into account our organisational and financial limitations, as well as expected estimates for the means and standard deviations that were obtained in previous studies. Additionally, we used the G* power software to calculate the total sample size of participants. The minimum sample size was 32 subjects considering the Wilcoxon test for paired samples (two groups) with a 5% level of significance, statistical power of 85%, and an average effect size of 0.5. An excess of about 10% was considered to account for possible dropouts.

### Inclusion and exclusion criteria

Included patients were diagnosed with at least one of the disorders classified in group II of masticatory muscle disorders according to the DC/TMD [4, 22], namely muscle pain, contracture, hypertrophy, neoplasm, movement disorders and masticatory muscle pain attributed to systemic/central pain disorders. Exclusion criteria were the following: pharmacological treatment that can alter muscle tonus and/or reduce muscle pain (e.g. muscle relaxants, selective serotonin reuptake inhibitors, painkillers); systemic diseases, including neurological, oncological and hormonal, that can alter muscle tonus; age over 60; severe psychiatric conditions; no consent for participation in the study and other TMD treatments.

### Study procedure

At the baseline and final visit, each patient was examined according to DC/TMD [2] using the DC/TMD international examination form, had elastography examination and filled in the questionnaires. All patients were instructed about the nature of TMD, risk factors and healthy lifestyle. In addition, impressions necessary to produce the splints were taken during the baseline visit. The patients used occlusal splints for eight weeks; manual therapy started one week later and continued for seven weeks. The rationale for choosing such study period was dictated by organizational and funding limitations, long-term institutional experience in treating patient with TMD, and results of other research that showed improvement after several weeks of treating TMD [23–25].

**Fig. 1:**
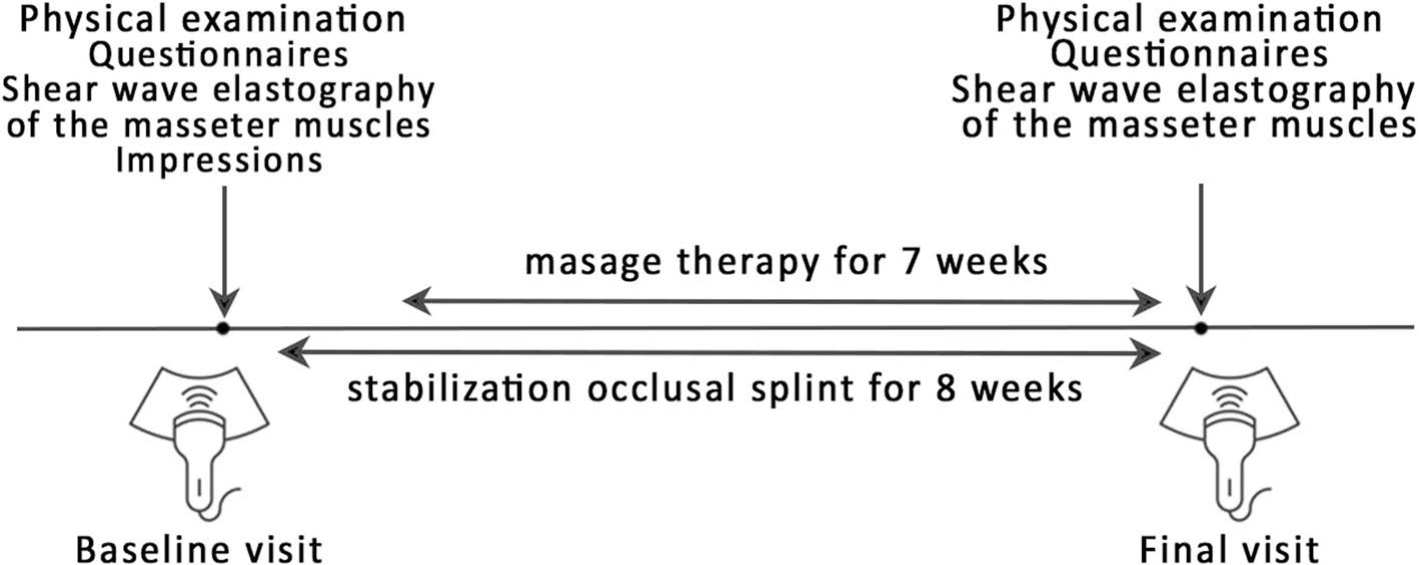
Study design

Figure 1 illustrates the study protocol.

### Intervention

Each patient received an individually produced lower stabilization occlusal splint covering the whole mandibular teeth arch with a thickness of 3 mm. The base of the splint was made of Erkodur (Erkodent, Pfalzgrafenweiler, Germany) while the shaft was made of DURASPLINT® LC (Scheu, Iserlohn, Germany). The patients were advised to use the splint during sleep and partly during the day for 12 h a day in total.

Each patient underwent seven physical therapy treatments: one 45-min session per week. The therapy involved the masseter, temporal, medial pterygoid and suboccipital muscles. The suboccipital muscles were treated because of their neurophysiological connection with the trigeminocervical nucleus and consequently facial pain [26]. The therapeutic intervention consisted of extra- and intraoral techniques. All intraoral methods were applied using nitrile gloves. Therapy included kneading, transverse friction movements, stretching and releasing the trigger points. Kneading involved circular motions performed by a therapist in the area of certain muscles. It starts the therapy and increases the blood level and temperature of tissues [27]. Transverse friction movements were executed with the pressure of the fingertips on the muscle belly and by moving perpendicular to the muscle fibres. It may provoke a local inflammatory response and then reconstruction of the muscle structure [27]. Stretching was done with the patient’s active opening and closing of the jaw while putting pressure on muscle fibres. This technique was applied for temporal and masseter muscles. It provides a greater range of motion and lowers muscle tension [28, 29]. Releasing the trigger points was done by finding tender points in the muscles and then applying compressive force until the pain completely disappeared. The masseter relaxation was achieved intraorally by grabbing the muscle belly with the thumb (from inside) and the index finger from the outside. The other muscles were relaxed extraorally. Therapy was conducted by a physiotherapist with six years of experience in masticatory muscle physiotherapy.

### Numerical pain assessment and questionnaires

For the present study, we selected a battery of questionnaires that can be useful for the psychosocial assessment of TMD patients. We used a numerical rating scale (NRS) to evaluate pain intensity and Generalized Anxiety Disorder-7 (GAD-7) for anxiety. In addition, the patients were asked to fill in the Epworth Sleepiness Scale (ESS), Satisfaction with Life Scale (SWLS), Perceived Stress Scale (PSS) and the Somatic Symptom Scale-8 (SSS-8).

A numerical rating scale (NSR) is a standard tool for assessing pain in TMD patients [30]. We used a 10-point scale that produces results from 0 for no pain to 10 for the worst imaginable pain. In our study, it was used to evaluate six locations, namely the masseter and temporal muscles and the temporal muscle tendons on both sides.

The PSS-10 [31] is a 10-item questionnaire with a total score from 0 to 40. The version adapted and translated by Juczyński and Ogińska-Bulik [32] was employed to measure the perceived life stress among the respondents (the higher the score, the higher the level of stress). The scores ranging from 0 to 13 indicate low, from 14 to 26 moderate and from 27 to 40 high perceived stress. The GAD-7 [33] was developed to evaluate anxiety symptoms. All seven questions are scored on a 4-point Likert scale and can produce a total score ranging from 0 to 21, with higher scores denoting greater severity of measured disorders. For a score of 10 or greater, further evaluation is recommended. The scores below 5 represent minimal, 5–9 mild, 10–14 moderate and over 14 severe anxiety.

The Pittsburgh Sleep Quality Index (PSQI) [34] evaluates the quality of sleep over the past month. Nineteen questions grouped in seven components inform about sleep duration, disturbance, latency, daytime dysfunction due to sleepiness, sleep efficiency, overall sleep quality and sleep medication use. The global PSQI score can range from 0 to 21. The higher scores indicate worse sleep quality, with the cut-off > 5 defining poor sleepers.

The ESS [35] was created to measure daytime sleepiness. The scale consists of 8 items that can produce scores from 0 (indicates ‘would never nod off’) to 3 (a strong chance of nodding off). The individual results of each item are summed, and the total score can range from 0 and 24. Higher scores indicate a higher level of daytime sleepiness. The scores from 0 to 10 denote normal sleepiness in healthy adults, 11–14 indicate mild, 15–17 moderate and 18–24 severe sleepiness.

The SWLS [36, 37] is a short instrument developed to evaluate perceived satisfaction with one’s life in terms of global life satisfaction. The scale consists of 5 items that can give scores from 1 (strongly disagree) to 7 (strongly agree). The total score ranges from 5 to 35. The scores from 5 to 9 mean extreme dissatisfaction with life, 10–14 – dissatisfaction, 15–19 – slightly below average, 20–24 – average, 25–29 – satisfaction and 30–35 – high satisfaction. For both time points, only one person achieved scores below average.

The SSS-8 [38] is a brief measure of somatic symptom burden. It is an abbreviated version of the PHQ-15. The scale consists of 8 items that can give scores from 0 (not bothered at all) to 4 (very much bothered). Somatic problems are classified into no to minimal (0–3), low (4–7), medium (8–11), high (12–15) and very high (16–32).

### Share wave elastography

Elastography was performed using an Aixplorer Ultimate device (SuperSonic Imagine, Aix-en-Provence, France) with a high-frequency linear probe SL 18–5 (5–18 MHz). The examinations were carried out in a supine, relaxed and comfortable position. The patients were asked not to bite down or swallow. The probe used minimal pressure on the examined tissues. The ultrasound probe was placed parallel to the long axis of the masseter in the middle of the muscle belly, where the volume of the fibres is the highest. The probe placement and patient position are based on previous research [13, 39]. The measurements were taken at the widest and thickest part of the muscle belly [40, 41]. A circular region of interest (ROI) of 4 mm was selected. Three measurements of each muscle were made and then averaged for further analysis. We reported inter- and intraobserver agreements of the measurements taken with the Aixplorer Ultimate device in the previous publication. The stiffness of the masseter muscle was rated excellent and confirmed diagnostic accuracy of shear wave elastography [18]. A radiologist with eight years of experience evaluated the masseter stiffness using shear wave elastography.

### Statistical analysis

The data were statistically analysed. The Shapiro–Wilk test was used to test for normal distribution. The paired Student’s t-test was employed to compare the measurements before and after the treatment for variables with normal distribution. For those with distribution other than normal, the Wilcoxon test for paired samples was applied. Differences were considered statistically significant at a *p*-value of 0 < 0.05. Statistical analysis was done with MedCalc v. 19.5.3 (MedCalc Software Ltd., Ostend, Belgium).

## Results

### Participants

Of the 35 generally healthy Caucasian participants, 11 were male and 24 female. Their age ranged from 19 to 58 (mean age: 29.80 ± 8.54). A diagnosis of group II of masticatory muscle disorders according to the DC/TMD [4, 22] was made in the case of all patients included. None of the patients had  previous occlusal splint therapy, nor masticatory muscle physical therapy. All participants did not have any serious abnormalities in dentition and they completed the study protocol.

### Numerical pain assessment and questionnaires

The pain assessment conducted with the NRS showed that treatment eliminated pain almost entirely at each measured site. At baseline, the median scores ranged from 5 to 8; after treatment, they ranged from 0 to 1. All differences were statistically significant for *p*-values below 0.0001.

The patients’ subjective feelings about the perceived stress and anxiety were assessed using validated questionnaires. The results of PSS-10 showed that the patients experienced moderate stress with a trend towards low stress after treatment. The PSS-10 score dropped by 0.86. This drop was significant. The results of GAD-7 showed that the mean score was below clinical significance (below 10), so they did not require further diagnosis. The scores indicated mild anxiety. At baseline, seven individuals reported a score of 10 or greater, while five individuals exceeded this cut-off value after treatment. The total mean score dropped significantly (by 0.91) in response to treatment.

Sleep was assessed with the PSQI and ESS. According to the PSQI, the study group suffered from poor sleep quality (at 2 time points, the global score was over 5), although it decreased significantly (by 0.54). According to the ESS, daytime sleepiness decreased significantly (by 0.94 points), but both measurements produced results within the normal range. A score over 10 was reported by four individuals at baseline and by two after treatment.

Life satisfaction measured by the SWLS indicated that the mean results leaned into a high score. The mean total score increased significantly (by 0.57 points). The SSS-8 showed that somatic problems dropped significantly from medium (9 points) to low (4 points). Only four patients reported no to minimal problems at baseline, but this number grew to 12 after treatment.

The results of PROMs are presented in Table 1.

**Table 1:** Results of numerical pain assessment and questionnaire scores

Muscle / Scale	Before	After	*p*-value
NRS, right masseter, median (IQR)	7 (5.25–7.00)	1 (1.00–1.75)	< 0.0001*
NRS, left masseter, median (IQR)	6 (5.00–7.00)	1 (1.00–2.00)	< 0.0001*
NRS, right temporal muscle, median (IQR)	5 (3.00–6.00)	1 (0.00–1.00)	< 0.0001*
NRS, left temporal muscle, median (IQR)	5 (3.00–6.00)	0 (0.00–1.00)	< 0.0001*
NSR, right temporal muscle tendon, median (IQR)	8 (7.00–8.00)	1 (1.002–.00)	< 0.0001*
NSR, left temporal muscle tendon, median (IQR)	8 (7.00–8.00)	1 (1.00–2.00)	< 0.0001*
PSS-10, mean ± SD	14.66 ± 6.06	13.80 ± 5.94	0.0917**
GAD-7, mean ± SD	6.71 ± 3.43	5.80 ± 3.26	0.0003**
PSQI, mean ± SD	7.83 ± 2.43	7.29 ± 2.22	0.0097**
ESS, mean ± SD	6.17 ± 3.08	5.23 ± 2.96	< 0.0001**
SWLS, mean ± SD	26.69 ± 3.84	27.26 ± 3.85	0.0008**
SSS-8, median (IQR)	9 (6.25–12.00)	4 (3.00–5.75)	< 0.0001*

*ESS* Epworth Sleepiness Scale, *GAD-7* Generalized Anxiety Disorder-7, *IQR* Interquartile range, *NRS* numerical rating scale, *PSQI* Pittsburgh Sleep Quality Index, *PSS* Perceived Stress Scale, *SD* Standard deviation, *SSS-8* Somatic Symptom Scale-8 (SSS-8), *SWLS* Satisfaction with Life Scale

^*^Wilcoxon test for paired samples

^**^paired samples t-test

### Share wave elastography

The stiffness of both masseter muscles decreased significantly (by 4.21 kPa on average) (Table 2). In two patients, the stiffness did not drop or increase, and the patients did not report improvement in symptoms. Both were referred for further diagnosis; one patient was diagnosed with tetany, no follow-up information is available for the other patient.

**Table 2:** Share wave elastography results for the masseter

Measured site	Before	After	*p*-value
Right masseter (kPa), mean ± SD	12.17 ± 1.16	7.90 ± 1.73	< 0.0001*
Left masseter (kPa), mean ± SD	12.18 ± 1.18	8.01 ± 1.69	< 0.0001*
Total measurements (kPa), mean ± SD	12.17 ± 1.17	7.96 ± 1.70	< 0.0001*

*SD* Standard deviation

^*^Wilcoxon test for paired samples

### Correlations between the change in PROMs and masseter stiffness

First, we calculated mean changes in somatic and mental measures and the masseter stiffness achieved in response to applied treatment. The total mean reduction in stiffness after the treatment in comparison with the baseline was 4.22 ± 1.47 kPa. The relationships between scales and the reduction in stiffness were not significant (Table 3). The correlation between baseline subjective measure scores and stiffness for both sides was significant or tended to be significant only for GAD (left: *r* = -0.3725; *p* = 0.0275; right: *r* = -0.333; *p* = 0.0506).

**Table 3:** Relationships between the changes in stiffness, numerical pain assessment and questionnaires scores

Muscle / Scale	Difference, mean ± SD	Correlation with stiffness, correlation coef.; *p*-value	
		right masseter	left masseter
Stiffness in the right masseter	4.17 ± 1.47		
Stiffness in the left masseter	4.26 ± 1.48		
NRS for the right masseter	5.14 ± 1.52	0.1461; 0.4024	
NRS for the left masseter	5.00 ± 1.5		0.4396; 0.0082
PSS-10	0.86 ± 2.92	-0.0917; 0.6002	-0.0511; 0.7708
GAD-7	0.91 ± 1.36	-0.1746; 0.3158	-0.1412; 0.4185
PSQI	0.54 ± 1.17	0.1195; 0.4942	0.0212; 0.9038
ESS	0.94 ± 1.11	0.1063; 0.5434	0.1003; 0.5663
SWLS	-0.57 ± 0.92	-0.0660; 0.7063	-0.1628; 0.3501
SSS-8	5.29 ± 4.29	-0.0201; 0.9086	-0.0088; 0.9602

*ESS* Epworth Sleepiness Scale, *GAD-7* Generalized Anxiety Disorder-7, *IQR* Interquartile range, *NRS* numerical rating scale, *PSQI* Pittsburgh Sleep Quality Index, *PSS* Perceived Stress Scale, *SD* Standard deviation, *SSS-8* Somatic Symptom Scale-8 (SSS-8), *SWLS* Satisfaction with Life Scale

## Discussion

Our study showed a significant improvement after masticatory muscle disorders treatment. Improvement in numerical pain assessment and questionnaire scores and decreased masseter stiffness were observed. An improvement in subjective PROMs, including perceived pain, anxiety, stress, quality of sleep, satisfaction with life and perceived somatic symptoms, corresponds to the reduction of stiffness of the masseter muscle as objectively measured with shear wave elastography.

TMD therapy is challenging. TMD is an umbrella term that includes various conditions affecting the morphology and function of the masticatory system. On top of this, numerous factors complicate the diagnosis and treatment of TMD, including those related to dentition, teeth grinding/clenching, systemic diseases and local neuromuscular pathologies [42]. TMD treatment aims to eliminate pain, joint sounds and improve mouth opening and jaw movements. Conservative therapies include interventions directed to change lifestyle (mainly to reduce stress), physiotherapy and occlusal splints [43]. In this study, we used conservative treatment, as it is proved to be effective, less aggressive and satisfactory for patients [42]. Treatment monitoring is based mostly on the physical examination and PROMs, which are subjective in nature. Currently, shear wave elastography is not applied to assess the masseter muscle stiffness in TMD patients. However, several reports proved its usefulness in evaluating the masseter muscle condition both in healthy volunteers and patients with diagnosed TMD [18, 19, 21, 44]. Still, there was a gap in evidence on monitoring TMD patients with shear wave elastography. Previous reports showed effectiveness to detect improvement after short term exposure to massage or exercise [19, 21]. After such treatment, the stiffness of the masseter muscle dropped from 12.17 ± 1.17 kPa to 7.96 ± 1.70 kPa (*p* < 0.0001). The present study showed a significant decrease in masseter muscle stiffness by 4.22 ± 1.47 kPa on average. This study justifies the use of shear wave elastography to assess long-term treatment.

As shear wave elastography can detect changes in the stiffness of the masseter with high precision, we aimed to investigate whether the benefits of manual therapy and stabilization occlusal splint in the treatment of masticatory muscle disorders can be shown using shear wave elastography. Correlations between the change in stiffness and PROM scores were not significant, which indicates that clinical drop in muscle stiffness corresponded to the improvement reported by patients in subjective measures. We did not find similar studies involving shear wave elastography in the literature, yet previous research showed that both massage and occlusal splints can effectively reduce masseter muscle stiffness. Researchers conclude that the drop in stiffness after massage [19, 20] can be due to relaxation of the tissues caused by increased blood flow and temperature of the treated tissue [45]; however, the biomechanism of massage has not been explained. Occlusal splints are widely used for treating TMD in patients with muscle-related disorders, although the evidence might be considered inconclusive. Occlusal splints reduce fatigue in the masseter muscles, ensure stable dental occlusion, reduce occlusal interferences, and minimise neuromuscular activity [46].

Benefits provided by shear wave elastography for TMD patients go beyond measuring stiffness and monitoring the treatment results. In the present study, we were able to identify two patients who did not respond to treatment (one completely, one partially). Although the patients reported a minimal clinical improvement, the masseter muscle stiffness increased in both. This finding prompted us to refer non-responsive patients for further diagnosis. Both patients were diagnosed with tetany. Tetany involves muscle contractions, but its aetiology is different and associated with hypocalcaemia [47]. Also, tetany has a variable clinical presentation, which poses a challenge for differential diagnosis [48]. We did not track down any report describing tetany in patients undergoing TMD treatment; however, this could be due to the lack of sensitive methods to distinguish those disorders. Using shear wave elastography, we could objectively measure that those patients had no improvement in terms of masseter muscle stiffness which allowed us to conclude that they were misdiagnosed. The patients were unaware of the condition at the time of enrolment in the study.

### Limitations

Several limitations have to be kept in mind when interpreting the study findings. First, this study included a relatively small number of subjects. The study offered an intensive treatment; therefore, only highly motivated and committed patients could participate. Second, the study lacks a control group. It would add more value to our results; however, we considered it ethically controversial to leave patients suffering from TMD without treatment and merely observe them. And finally, our study lasted eight weeks including seven weeks of massage therapy and eight week od occlusal splints wearing only without a long-term follow-up due to financial, organizational and time constraints. Nevertheless, it was sufficient for a short-term assessment that showed the benefits of manual therapy and the application of stabilization occlusal splint for masticatory muscle disorders treatment. However, further questions about maintenance therapy and the possibility of relapse after treatment remain open.

## Conclusions

Conservative therapy of masticatory muscle disorders involving manual therapy and stabilization occlusal splint is effective. It reduces the masseter stiffness as objectively shown in shear wave elastography and improves subjective PROMs scores, including numerical pain assessment and selected questionnaires. Shear wave elastography has the potential for broad application in clinical practice to monitor a masticatory muscle disorders treatment effects due to its objectivity and non-invasive character. Further research is recommended on larger patient populations and longer follow-up.

## Supplementary Information

**Additional file 1.**

## Data Availability

All data generated or analysed during this study are included in this published article and its supplementary information file.

## Abbreviations

ESS: Epworth Sleepiness Scale
GAD-7: Generalized Anxiety Disorder-7
IQR: Interquartile range
NRS: Numerical rating scale
PSQI: Pittsburgh Sleep Quality Index
PROM: Patient-reported outcome measure
PSS: Perceived Stress Scale
SD: Standard deviation
SSS-8: Somatic Symptom Scale-8 (SSS-8)
SWLS: Satisfaction with Life Scale
TMD: Temporomandibular disorders
